# Acetate Affects the Process of Lipid Metabolism in Rabbit Liver, Skeletal Muscle and Adipose Tissue

**DOI:** 10.3390/ani9100799

**Published:** 2019-10-14

**Authors:** Lei Liu, Chunyan Fu, Fuchang Li

**Affiliations:** Key Laboratory of Animal Biotechnology and Disease Control and Prevention of Shandong province, Department of Animal Science, Shandong Agricultural University, Shandong 271018, China; leiliu@sdau.edu.cn (L.L.); fuchunyan1004@126.com (C.F.)

**Keywords:** acetate, lipid metabolism, signaling pathway, rabbit

## Abstract

**Simple Summary:**

Lots of short-chain fatty acids (SCFAs) are produced in the rabbit cecum after dietary fiber fermentation. In addition to supplying energy, SCFAs could regulate lipid metabolism, but the related mechanism is still unknown. In our experiment, we study the effect of acetate (major SCFAs, 70–80%) on rabbit lipid metabolism. The present study found that acetate alters the process of lipid metabolism in rabbit liver, skeletal muscle and adipose tissue, and inferred some signaling pathways related to the process. A mechanism of acetate-regulating lipid metabolism is useful to identify the function in fat metabolism of microbiological products from rabbit and rabbit processes for nutrition metabolism.

**Abstract:**

Short-chain fatty acids (SCFAs) (a microbial fermentation production in the rabbit gut) have an important role in many physiological processes, which may be related to the reduced body fat of rabbits. In the present experiment, we study the function of acetate (a major SCFA in the rabbit gut) on fat metabolism. Ninety rabbits (40 days of age) were randomly divided into three groups: a sham control group (injection of saline for four days); a group experiencing subcutaneous injection of acetate for four days (2 g/kg BM per day, one injection each day, acetate); and a pair-fed sham treatment group. The results show that acetate-inhibited lipid accumulation by promoting lipolysis and fatty acid oxidation and inhibiting fatty acid synthesis. Activated G protein-coupled receptor 41/43, adenosine monophosphate activated protein kinase (AMPK) and extracellular-signal-regulated kinase (ERK) 1/2 signal pathways were likely to participate in the regulation in lipid accumulation of acetate. Acetate reduced hepatic triglyceride content by inhibiting fatty acid synthesis, enhancing fatty acid oxidation and lipid output. Inhibited peroxisome proliferator-activated receptor α (*PPARα*) and activated AMPK and ERK1/2 signal pathways were related to the process in liver. Acetate reduced intramuscular triglyceride level via increasing fatty acid uptake and fatty acid oxidation. PPARα was associated with the acetate-reduced intracellular fat content.

## 1. Introduction

In mammals, adipose is the major tissue of fat deposition. Fatty acids may be synthesized in adipose tissue, released into circulation, and delivered to other tissues. In muscle tissue, fatty acids are an important substrate for oxidation to supply energy. The liver is a center of fatty acid metabolism and lipid circulation via lipoprotein synthesis [1]. Hepatocytes accumulation of lipid droplets is a consequence of alterations in synthesis and oxidation of fatty acids and very low-density lipoprotein (VLDL) secretion [2].

Lots of factors are involved in the metabolism of fatty acids. Carnitine palmitoyl transferase 1 (*CPT1*) and fatty acid synthase (*FAS*) are respectively the rate-limiting enzymes of fatty acid oxidation and synthesis [3]. Hormone-sensitive lipase (HSL) hydrolyzes triglyceride (TG) in adipose tissue [4]. Lipoprotein lipase (LPL) catalyzes the hydrolysis of triglycerides in skeletal muscle [5]. Fatty acid transport protein (FATP) and fatty acid-binding protein (FABP) could promote cellular fatty acids uptake and transport [6]. Some signaling pathways are also associated with the regulation of fatty acids metabolism. The activation of adenosine monophosphate activated protein kinase (*AMPK*) increases fatty acid oxidation in skeletal muscle, inhibits fatty acid and cholesterol synthesis in liver and lipogenesis process in adipose tissue [7]. Peroxisome proliferator-activated receptor α (*PPARα*) has a high expression level in liver, adipose and skeletal muscle tissues, which is also involved in fatty acid metabolism [8]. *PPARγ* expression sites are mainly in adipose tissue, macrophage and mammary tissue [8,9]. In adipose tissue, *PPARγ*, as a transcriptional regulatory factor, could regulate various genes expression related to lipid metabolism (e.g., stearoyl-CoA synthetase and acyl-CoA synthetase) [10]. In addition, mitogen-activated protein kinases (MAPKs) comprise a family of related serine/threonine protein kinases, such as c-Jun NH2-terminal kinases (JNK), extracellular-signal-regulated kinase (ERK), and p38 MAPK, which play pivotal roles in many essential cellular processes. Recent studies show that ERK could regulate the adipogenic process [11]. A p38 inhibitor treatment decreases the process of adipocyte differentiation in 3T3-L1 [12]. The inhibition of JNK signal attenuates the high-fat diet-induced obesity [13,14]. The mammalian target of rapamycin (mTOR), a serine/threonine kinase, is involved in regulating various cells metabolism and development. Rapamycin, a mTOR inhibitor, inhibits strongly the adipogenic process in mouse adipocyte by affecting the clonal expansion of pre-adipocytes [15,16], which suggests that mTOR is necessary for adipogenic program in mammals [17].

Lots of short-chain fatty acids (SCFAs) are produced in the rabbit cecum after dietary fiber fermentation [18]. In addition to supplying energy, SCFAs have been known to have regulatory effects on insulin and glucagon secretion, gastrointestinal motility, and cell proliferation and differentiation [19,20,21,22]. Previous studies showed that acetate (a major SCFA, 70–80%) was a substrate for lipogenesis and stimulated adipocyte differentiation [23]. However, more studies are needed to clarify the regulatory mechanism of acetate on lipid metabolism. In the present study, we investigate the effect of acetate on the lipid metabolism and related signaling pathways in rabbit skeletal muscle, liver and adipose tissues and determine the possible pathways related to the regulation of acetate on lipid metabolism in rabbits.

## 2. Materials and Methods

### 2.1. Experimental Protocol and Sample Collection

At 40 days of age, 90 Hyla rabbits of similar body weight (1513 ± 10 g) were divided into three groups (30 rabbits per group): a sham treatment group (injection of saline at 8:00 AM, one injection each day, control); a group that experienced subcutaneous injection of acetate for four days at 8:00 AM, (2 g/kg of body weight per day, one injection each day, acetate) [24]; and a pair-fed sham treatment group with feed consumption equivalent to that of the acetate-treated rabbits on the previous day (pair-fed). Rabbits were individually housed in self-made cages (60 × 40 × 40 cm). Temperature was maintained at 20–23 °C. A light regime of 12 (light)/12 (dark) was applied. The diets were formulated according to the recommendation of de Blas and Mateos [25], and the feed was made into 4 mm pellets by using pressure. All rabbits received a starter diet containing 17% crude protein, 18% crude fiber and 10.55 MJ/kg of digestible energy. All rabbits had free access to feed and water during the rearing period. Body weight and feed intake were recorded daily during the period of experiment. All study procedures were approved by the Shandong Agricultural University Animal Care and Use Committee (SDAUA-2018-059) and were in accordance with the Guidelines for Experimental Animals established by the Ministry of Science and Technology (Beijing, China). Rabbits received the first injection at the age of 40 days and the last injection at the age of 43 days. After the last injection, eight rabbits from each group were fasted for 4 h, and then 5 mL of blood was collected from the heart of each fasted rabbit. Plasma was obtained after centrifugation at 380 g for 10 min at 4 °C and stored at −25 °C. These eight rabbits were sacrificed by cervical dislocation, and the liver, foreleg muscle, hindleg muscle, longissimus dorsi muscle, scapular fat and perirenal fat were weighed and collected. After being snap-frozen in liquid nitrogen, these tissue samples were stored at −80 °C.

### 2.2. Measurements

Plasma VLDL concentration was determined using the method described by Barter and Lally [26]. Tissues were disrupted and homogenized in isopropanol (1 g samples: 9 mL isopropanol). After centrifuging at a speed of 8000 g for 10 min, the supernatant was isolated. TG concentration in tissues and plasma was measured spectrophotometrically using commercial diagnostic kits (Jiancheng Bioengineering Institute, Nanjing, China). The operational process of Western blotting and quantitative real time PCR fully followed the instructions in our previous description [27]. The primers’ sequences are listed in Table 1.

### 2.3. Statistical Analysis

All of the data collected were subjected to one-way ANOVA analysis with Tukey’s multiple comparison procedure as post hoc test. All statistical analyses were performed using SAS software (Version 9.1, SAS Institute, Cary, NC, USA). Data are presented as means ± standard error. For food intake and body weight gain, n = 30; for ratio and yield of tissue or organ, TG and VLDL concentrations, gene and protein expression, n = 8. Means were considered significant at *p* < 0.05.

## 3. Results

### 3.1. Effect of Acetate on Rabbit Performance

**Figure 1:**
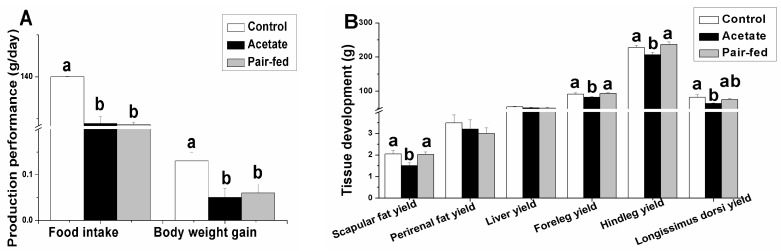
The effect of acetate administration on production performance (**A**) and tissue development (**B**). a, b: means with different superscripts are significantly different (*p* < 0.05).

Acetate administration significantly decreased daily feed intake, body weight gain and longissimus dorsi muscle yield compared with the control (Figure 1, *p* < 0.05). Acetate administration significantly decreased the scapular fat yield, foreleg yield and hindleg yield compared with the control and pair-fed counterparts (*p* < 0.05) while having no effect on the perirenal fat yield and liver yield (*p* > 0.05).

### 3.2. Effect of Acetate on TG and VLDL Concentrations in Tissue or Plasma

**Figure 2:**
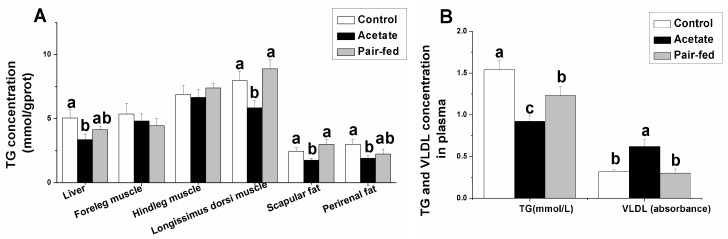
The effect of acetate treatment on TG concentration in tissue (mmol/gprot) (**A**) and TG (mmol/L) and VLDL concentration (absorbance) in plasma (**B**). a, b, c: means with different superscripts are significantly different (*p* < 0.05). (Abbreviations: TG, triglycerides; VLDL, very low-density lipoprotein.).

The concentrations of triglyceride in plasma, longissimus dorsi muscle and scapular fat and cholesterol in plasma were lower in acetate-treated group than in control and pair-fed groups (Figure 2, *p* < 0.05), while the changes in concentrations of triglyceride in foreleg and hindleg muscles were not significant (*p* > 0.05). Compared with the control, acetate decreased the triglyceride levels in liver and perirenal fat (*p* < 0.05). Acetate treatment significantly increased the plasma VLDL concentration compared with the control and pair-fed groups (*p* < 0.05).

### 3.3. Effect of Acetate on Genes Expression Related Lipid Metabolism in Liver, Skeletal Muscle and Adipose Tissue

**Figure 3:**
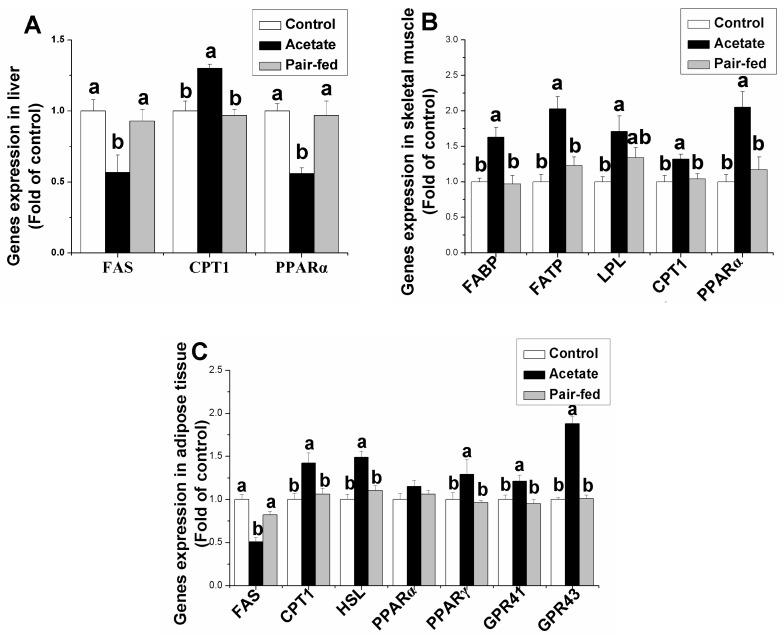
The effect of the acetate treatment on genes expression related to lipid metabolism in liver (**A**), skeletal muscle (**B**) and adipose tissue (**C**). a, b: means with different superscripts are significantly different (*p* < 0.05). (Abbreviations: FAS, fatty acid synthase; *CPT1*, carnitine palmitoyl transferase 1; *HSL*, hormone-sensitive lipase; *LPL*, lipoprotein lipase; *FATP*, fatty acid transport protein; *FABP*, fatty acid-binding protein; *PPAR*, peroxisome proliferator-activated receptor; *GPR*, G protein-coupled receptor.).

As shown in Figure 3, the *FAS* and *PPARα* mRNA levels in liver decreased following the acetate treatment compared with the control and pair-fed rabbits (*p* < 0.05). In contrast to the negative effect of acetate on *FAS* and *PPARα* genes expression, the acetate induced an increase in *CPT1* gene expression in liver (*p* < 0.05). In skeletal muscle, the acetate significantly increased the mRNA levels of *FABP*, *FATP*, *CPT1* and *PPARα* compared with the control and pair-fed groups (*p* < 0.05). Compared with the control, the acetate injection significantly increased the LPL gene expression in skeletal muscle (*p* < 0.05). In adipose tissue, the acetate treatment significantly decreased the genes expression of *FAS* and *LPL* compared with the control and pair-fed rabbits (*p* < 0.05) but significantly increased the genes expression of *CPT1*, *HSL*, *PPARγ*, G protein-coupled receptor (*GPR*) 41 and *GPR43* (*p* < 0.05). The acetate treatment had no significant effect on *PPARα* mRNA levels in adipose tissue (*p* > 0.05).

### 3.4. Effect of Acetate on Protein Expression Related Lipid Metabolism in Liver, Skeletal Muscle and Adipose Tissue

**Figure 4:**
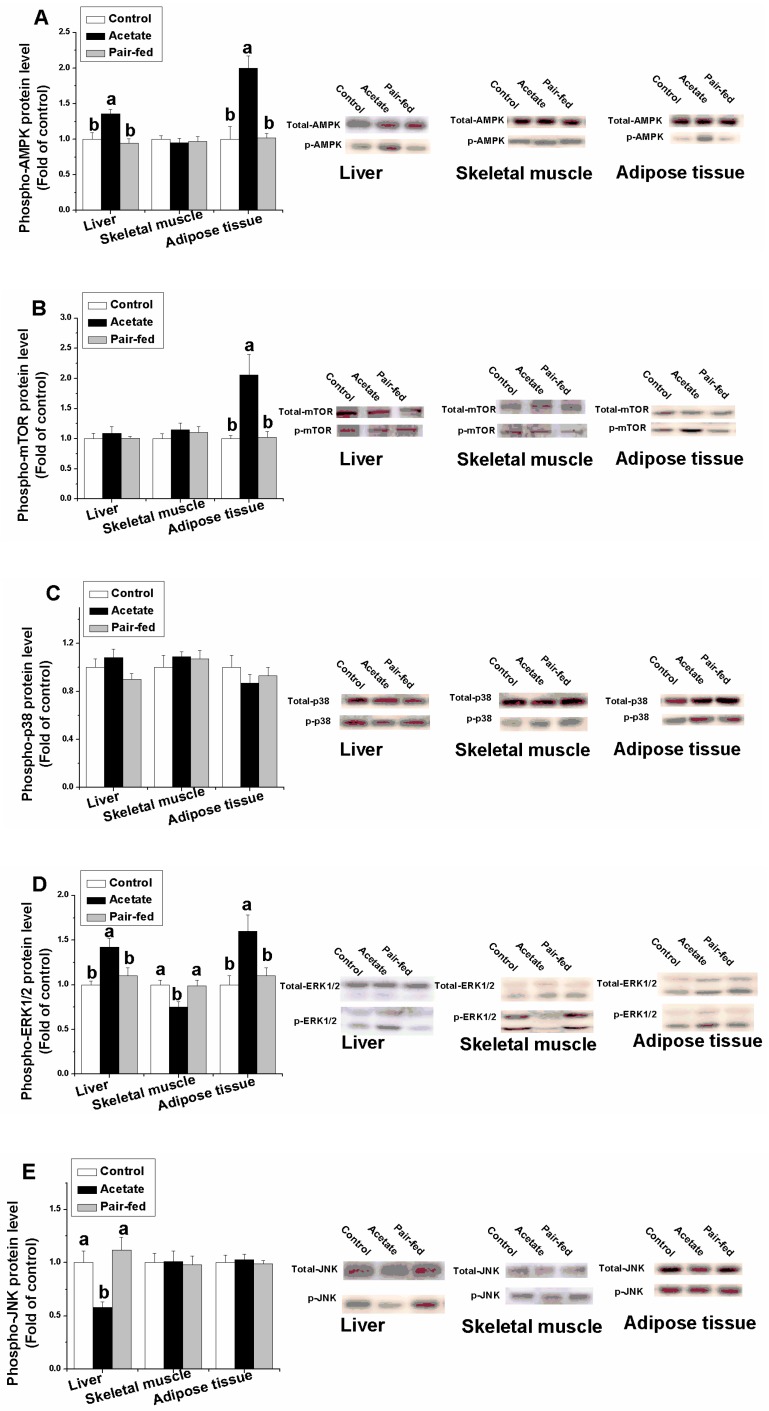
The effect of acetate treatment on protein expression of AMPK (**A**), mTOR (**B**), p38 (**C**), ERK1/2 (**D**) and JNK (**E**) in liver, skeletal muscle and adipose tissue. a, b: means with different superscripts are significantly different (*p* < 0.05). (Abbreviations: AMPK, adenosine monophosphate zctivated protein kinase; p38, p38 mitogen-activated protein kinase; JNK, c-Jun NH2-terminal kinases; ERK, extracellular-signal-regulated kinase; mTOR, mammalian target of rapamycin).

When compared with the control and pair-fed groups, acetate significantly increased the protein levels of p-AMPK in liver and adipose tissue, p-mTOR in adipose tissue, and p-ERK in liver and adipose tissue (Figure 4, *p* < 0.05), while significantly decreasing the protein levels of p-ERK in skeletal muscle and p-JNK in liver (*p* < 0.05). No significant changes were found in the protein levels of p-AMPK in skeletal muscle, p-mTOR in liver and skeletal muscle, p38 MAPK in liver, skeletal muscle and adipose tissue, or p-JNK in skeletal muscle and adipose tissue between the acetate-treated group and the control group (*p* > 0.05).

## 4. Discussion

We demonstrated the effect of acetate administration on fat metabolism in rabbits for the first time. Our data suggested that: (1) acetate-inhibited lipid accumulation promotes lipolysis and fatty acids oxidation and inhibits fatty acids synthesis; (2) acetate reduced the hepatic TG content by inhibiting fatty acid synthesis while enhancing fatty acid oxidation and lipid output; (3) acetate decreased intramuscular TG concentration via increasing the uptake and oxidation of fatty acids; and (4) AMPK and ERK1/2 signaling in liver, PPARα signaling in skeletal muscle, and *GPR41*/43, AMPK, mTOR and ERK1/2 signaling in adipose tissue were associated with acetate-regulated fat metabolism.

### 4.1. Acetate-Inhibited Lipid Accumulation

A previous study revealed that SCFAs mediate various physiological functions involved in homoeostasis [28]. In line with those studies [27], our study showed that acetate decreased the feed intake, body weight gain, and fat and muscle weight, which suggested an altered energy distribution that attenuates lipid deposition after acetate administration. The decrease in the absolute weight of adipose tissue is related to increased *HSL* gene expression. Studies showed that *HSL* could possess triacylglycerol lipase activity and appears to be the rate-limiting enzyme of cholesteryl ester and diacylglycerol hydrolysis in adipose tissue. [29]. Meanwhile, we found that acetate decreased the TG concentration of adipocyte, which is related to the alteration of *CPT1* and FAS genes expression. Decreased FAS mRNA levels after acetate treatment implies that acetate could inhibit fatty acid synthesis [30], and increased *CPT1* mRNA levels after acetate treatment suggest an enhanced mitochondrial uptake of fatty acyl-CoA for β-oxidation [31].

### 4.2. Acetate Reduced the Hepatic Lipid Content

The liver is a center of fatty acid metabolism and lipid circulation via lipoprotein synthesis in mammals. Fatty acids synthesis in liver is tightly controlled by nutritional conditions [32]. FAS gene expression was significantly decreased in liver after acetate treatment in our study, indicating that the process of hepatic fatty acids synthesis is significantly weakened by acetate. Meanwhile, up-regulated *CPT1* mRNA levels in the acetate group indicate an increase in the fatty acid catabolic process. VLDL is the major form of synthetic lipid in liver releasing into blood. In our study, acetate significantly increased the plasma VLDL concentration compared with the control and pair-fed groups, indicating that acetate promotes lipid output from the liver. The results suggest that acetate decreases hepatic TG content by inhibiting the fatty acid anabolic process and increasing fatty acid catabolism and output of triglycerides from liver. This result is in line with the previous studies in rats [33].

### 4.3. Acetate Decreased the Intramuscular Lipid Content

Fatty acids are an important fuel source for oxidative metabolism in muscles [34]. However, the research on regulation of acetate in lipid metabolism in muscle is scarce. Several physiological steps are involved in lipid uptake, transport and oxidation in muscle. FATP and FABP could promote the uptake and use of long-chain fatty acids [35]. Our study shows that acetate increased the genes expression of FATP and FABP in skeletal muscle, indicating that the process of fatty acids uptake in muscle cells may be increased, which subsequently causes the drastic decrease in plasma TG. In addition, our study shows that acetate increases lipolysis and fatty acid oxidation by up-regulating LPL and *CPT1* genes expression. Therefore, skeletal muscle could receive abundant energy supply. We also found that acetate decreased the intramuscular TG content, which implies that the increased speed after acetate treatment in lipid transport and oxidation was greater than the lipid uptake.

### 4.4. PPAR Signaling Was Associated with the Acetate-Regulated Lipid Metabolism

*PPARα*, a nuclear receptor, regulates lipid metabolism in liver and skeletal muscle. *PPARα* gene major expresses in peroxisome and mitochondrion and regulates the fatty acids β-oxidation and uptake, and lipoprotein assembly and transport [35,36]. *PPARα* deficiency in mice severely impairs fatty acid uptake and β-oxidation [36,37]. In humans and rats, *PPARα* could stimulate *CPT1* gene expression through a peroxisome proliferator response element (PPRE) [38]. In line with a previous study [39], *PPARα* gene expression showed a similar tendency to the *CPT1* gene in muscle after acetate treatment. Besides, the effect of acetate on PPARα gene expression occurs in a tissue-specific manner. Acetate increased the *PPARα* gene expression in muscle, decreased the PPARα gene expression in liver, and did not alter the *PPARα* gene expression in adipose tissue. The results indicate that acetate influences lipid metabolism in skeletal muscle, presumably through the *PPARα* signaling pathway, but not in adipose tissue. Additionally, we noted the opposite tendency of *PPARα* with *CPT1* gene expression and lipid output in liver after acetate treatment, indicating the complexity of the acetate regulation of *PPARα* gene expression. The function of the *PPARα* signaling pathway in acetate regulation of lipid metabolism in liver tissue needs further study.

*PPARγ*, highly expressed in both white adipose tissue and brown adipose tissue, could cause adipocyte differentiation and insulin sensitivity [40]. Our study shows that acetate up-regulated the *PPARγ* gene expression in adipose tissue, in line with previous research [41,42]. Couvigny et al. [41] found that SCFAs are linked to metabolic reprogramming and immune functions via up-regulating *PPARγ* gene expression in intestinal epithelial cells. Our results imply that acetate enhances the adipocyte differentiation via up-regulating *PPARγ* gene expression in adipose tissue [42].

### 4.5. MAPK Signaling Was Associated with the Acetate-Regulated Lipid Metabolism

MAPKs play key roles in many intracellular processes (e.g., proliferation and differentiation). Recent results showed ERK1/2 could regulate *PPARγ* gene expression and promote adipogenic differentiation of adipocytes [11,43]. Regulation of acetate in *PPARγ* transcription and adipocyte differentiation in adipose tissue may be associated with the ERK1/2 signal pathway. Menendez et al. [44] found that FAS gene inhibition could activate the ERK1/2 signal. Therefore, the opposite changes in FAS gene expression and p-ERK1/2 protein in liver and adipose tissue imply that ERK1/2 plays a key role in acetate-inhibited fatty acid synthesis. A recent study in adipocytes shows that *HSL* is a downstream target of ERK1/2 [45]. In our experiment, lipolysis stimulated by acetate may be via ERK1/2 signal pathway. In rodent skeletal muscle, ERK1/2 inhibition prevents the contraction-induced increase in plasma membrane FATP content and lipid uptake [46]. We could not confirm the function of ERK1/2 in acetate-regulated lipid use in skeletal muscle because acetate decreased the p-ERK1/2 protein in skeletal muscle, which is the opposite performance with the FATP and FABP genes expression.

Previous studies showed the regulatory role of p38 MAPK in lipid metabolism, but the results were inconsistent. Engelman et al. [12] found the positive role for p38 MAPK in adipogenesis, which may be related to the decreases in *PPARγ* and C/EBPβ gene expression. The inhibition of p38MAPK increases adipogenesis from embryonic to adult stages, which may be associated with the enhancing C/EBPβ transcriptional activity [47]. Meanwhile, the p38 MAPK chemical inhibitor treatment decreases bone morphogenetic protein 2-induced adipocyte formation, which is associated with decreases in *PPARγ* and *C/EBPβ* transactivation activities [48]. In our study, p38 MAPK protein was not altered by acetate compared with the control and pair-fed groups in liver, muscle and adipose tissues, which suggests that p38 MAPK may be not the major target in acetate-regulating lipid metabolism.

Obesity is associated with abnormally elevated JNK activity, and the absence of JNK results in substantial protection from obesity-induced insulin resistance [13]. JNK deficiency enhances fatty acid use in cultured myotubes [49]. Our study shows that acetate decreases the p-JNK protein levels in liver, which is in accordance with earlier research [50]. The result suggests that the JNK signal pathway is involved in the regulation of acetate in fatty acid use in liver but not in muscle and tissue.

### 4.6. GPR41/43 Signaling Pathway Was Associated with the Acetate-Regulated Lipid Metabolism in Adipose Tissue

*GPR41* and 43 are highly expressed in adipose tissue of pigs and mice [42,51]. In our study, we found also high expression of *GPR41* and 43 in adipose but did not detect the *GPR41* and *43* genes in liver and muscle, which is in agreement with the findings of Fu et al. [52]. In 3T3-L1 cells, *GPR43* blocking inhibited the *PPARγ* transcription [53]. Thus, *GPR41/43* might play an important role in lipid metabolism in adipose tissue. The effect of acetate in *GPR41/43* gene expression is species-specific. Hong et al. [53] found that acetate stimulated adipocyte differentiation in 3T3-L1 cells via *GPR43* but not *GPR41*. However, *GPR41/43* was not involved in acetate-regulated adipogenesis in porcine stromal-vascular fraction cells [42]. However, acetate increased significantly the *GPR41* and *43* genes expression in our experiment, which suggests that *GPR41* and 43 probably participate in the regulatory process of acetate in lipid metabolism in rabbit adipose tissue.

### 4.7. AMPK Signaling Pathway Was Associated with the Acetate-Regulated Lipid Metabolism in Adipose Tissue

AMPK, a crucial cellular energy sensor, could stimulate of fatty acid oxidation and restrain intracellular lipid accumulation [54]. AMPK could regulate the fatty acid synthesis process via repressing FAS gene expression, and also promote the β-oxidation process of long-chain fatty acids via activating *CPT1* [55,56]. SCFAs may indirectly regulate AMPK activity via oxidation to generate adenosine triphosphate and may also directly alter AMPK activity as a signal substance in some cells such as colonocytes [57]. To investigate the function of AMPK in acetate-regulating lipid metabolism, we measured p-AMPKα (Thr172), which is essential for AMPK activity [58]. We found that the effect of acetate on p-AMPK levels is tissue-specific that p-AMPKα levels increased after acetate administration in liver and adipose tissue but not in muscle tissue, indicating that AMPK signaling may be involved in the acetate-regulated fatty acid metabolism in liver and adipose tissue but not in muscle tissue.

### 4.8. mTOR Signaling Was Associated with the Acetate-Regulated Lipid Metabolism in Adipose Tissue

Recent research showed that the mTOR pathway plays an important role in the synthesis and secretion of triacylglycerol [59]. Inhibition of mTOR signaling reduced intracellular lipid accumulation in goose and fish hepatocytes and 3T3-L1 adipocytes [15,59,60]. In our study, we found a tissue-specific effect of acetate on mTOR protein expression. In rabbits, adipose tissue is more sensitive in response to acetate than liver and muscle tissues. mTOR controls the adipogenesis process by regulating the *PPARγ* gene expression [61]. The concurrent increase in mTOR protein and *PPARγ* gene expression after acetate exposure indicate that the mTOR signal may be related to acetate-caused adipocyte differentiation.

## 5. Conclusions

Acetate decreased the lipid synthesis and accumulation and increased the fatty acids use of skeletal muscle. AMPK, ERK1/2 signaling in liver, PPARα signaling in skeletal muscle, and GPR41/43, AMPK, mTOR and ERK1/2 signaling pathways in adipose tissue were associated with acetate-reduced intracellular fat content.

## Figures and Tables

**Table 1:** Rabbit primers information for gene expression.

Gene	Genebank Accession Number	Primers Sequences (5′–3′)	Product Size (bp)
*GAPDH*	NM_001082253	F: TGCCACCCACTCCTCTACCTTCG	163
		R: CCGGTGGTTTGAGGGCTCTTACT	
*β-actin*	NM_001101683.1	F:CGCAGAAACGAGACGAGATT	168
		R:GCAGAACTTTGGGGACTTTG	
*GPR41*	XM_002722237.2	F:CCATCTATCTCACCTCCCTGTTC	130
		R:AACCAGCAGAGCCCACTGAC	
*GPR43*	XM_002722218.2	F:CGTCCAACTTCCGCTGGTA	146
		R:CTTGTACTGCACGGGGTAGG	
*PPARγ*	NM_001082148.1	F:GGAGCAGAGCAAAGAAGTCG	111
		R:CTCACAAAGCCAGGGATGTT	
*FATP*	XM_002722970	F:GGCCTACCTCTCTGGTGATG	111
		R:TCAGTGGTGGACACGTTCTC	
*FABP*	XM_002716060	F:AGCTGGTGGACAGCAAGAAT	129
		R:TCAGGGTGATGATGTCTCCA	
*CPT1*	XM_002724092.2	F:ATTCTCACCGCTTTGGGAGG	196
		R:ACGGGGTTTTCTAGGAGCAC	
*FAS*	KF201292.1	F:ACCACGTCCAAGGAGAGCA	112
		R:AGTTCTGCACCGAGTTGAGC	
*HSL*	XM_008249691.2	F: CCAGGCTAAACTCGCATCCA	119
		R: ATTTGGCTCTCTGGACTGGC	
*LPL*	NM_001177330.1	F: TTCAACCACAGCAGCAAGAC	141
		R: TAACAGCCAGTCCACCACAA	
*PPARα*	XM_002723354	F:AGGCCCTCTTCAGAACCTGT	122
		R:GTGGCTTTCTGTTCCCAGAG	

Abbreviations: *FAS*, fatty acid synthase; *CPT1*, carnitine palmitoyl transferase 1; *HSL*, hormone-sensitive lipase; *LPL*, lipoprotein lipase; *FATP*, fatty acid transport protein; *FABP*, fatty acid-binding protein; *PPAR*, peroxisome proliferator-activated receptor; *GAPDH*, glyceraldehyde 3-phosphate dehydrogenase; *GPR*, G protein-coupled receptor.
